# Update on the Genetics of and Systemic Therapy Options for Combined Hepatocellular Cholangiocarcinoma

**DOI:** 10.3389/fonc.2020.570958

**Published:** 2020-09-25

**Authors:** Alexander A. Azizi, Andreas V. Hadjinicolaou, Carla Goncalves, Adam Duckworth, Bristi Basu

**Affiliations:** ^1^Addenbrooke's Hospital, Cambridge University Hospitals NHS Foundation Trust, Cambridge, United Kingdom; ^2^Department of Oncology, University of Cambridge, Cambridge, United Kingdom

**Keywords:** Combined hepatocellular-cholangiocarcinoma, cHCC-ICC, cHCC-CCA, biphenotypic, primary liver cancer, genomics, systemic therapy

## Abstract

Combined hepatocellular-cholangiocarcinoma (cHCC-ICC) is an uncommon and aggressive form of primary liver cancer. Currently, there are no international guidelines for optimal management. For localized tumors, radical resection represents the preferred treatment option, whereas for advanced tumors, systemic therapies recommended for intrahepatic cholangiocarcinoma (ICC) and hepatocellular carcinoma (HCC) are often selected. Emerging information from comparative cohort studies, genomic and transcriptomic data sets are starting to build a case for rationalized approaches to systemic treatment in the advanced setting specific to cHCC-ICC.

## Introduction

Combined hepatocellular-cholangiocarcinoma (cHCC-ICC/ cHCC-CCA) or “biphenotypic” primary liver cancer is a form of primary liver carcinoma (PLC) with phenotypic characteristics of both hepatocytic and cholangiocytic differentiation (1, 2). Additional acceptable terminology for this form of PLC is mixed hepatocellular-cholangiocarcinoma (mixed HCC-CC), mixed hepatobiliary carcinoma, or hepatocholangiocarcinoma (3). At present, there are no accepted international management guidelines; there is no standard first line systemic therapy option for cHCC-ICC and it has a dismal prognosis, worse than that of either hepatocellular carcinoma (HCC) or cholangiocarcinoma (CCA) (1, 4, 5). This review focuses on the genetics of and current systemic treatment options for advanced, unresectable and metastatic cHCC-ICC in order to provide a platform for future trials.

### Epidemiology

cHCC-ICC is likely to comprise between 0.4 and 4.7% of all PLCs, incidence ratio for male:female patients is 1.8–2.1:1 and median age at diagnosis is 62–65 years-old (2, 6–12). Data from the Surveillance, Epidemiology, and End Results (SEER) Program of the National Cancer Institute reveals that patients tend to present with distant, metastatic disease (130/380, 34.2%) rather than localized (98/380, 25.8%) or regional disease (97/380, 25.5%) according to their generic staging system (*vida infra*) (11, 12). The risk factors remain unclear and retrospective case-control studies report conflicting associations; some Asian studies suggest similarities between the risk factors for hepatocellular carcinoma (HCC) and cHCC-ICC such as chronic liver disease caused by infection with hepatotropic viruses such as hepatitis B (HBV) or hepatitis C (HBC) and alcohol. Western world datasets however propose closer similarities to the risk factors associated with intrahepatic cholangiocarcinoma (ICC) such as primary sclerosing cholangitis, chronic liver fluke infections, biliary-duct cysts, and hepatolithiasis (4, 10, 13–16).

### Histological Characterization and classification

**Figure 1:**
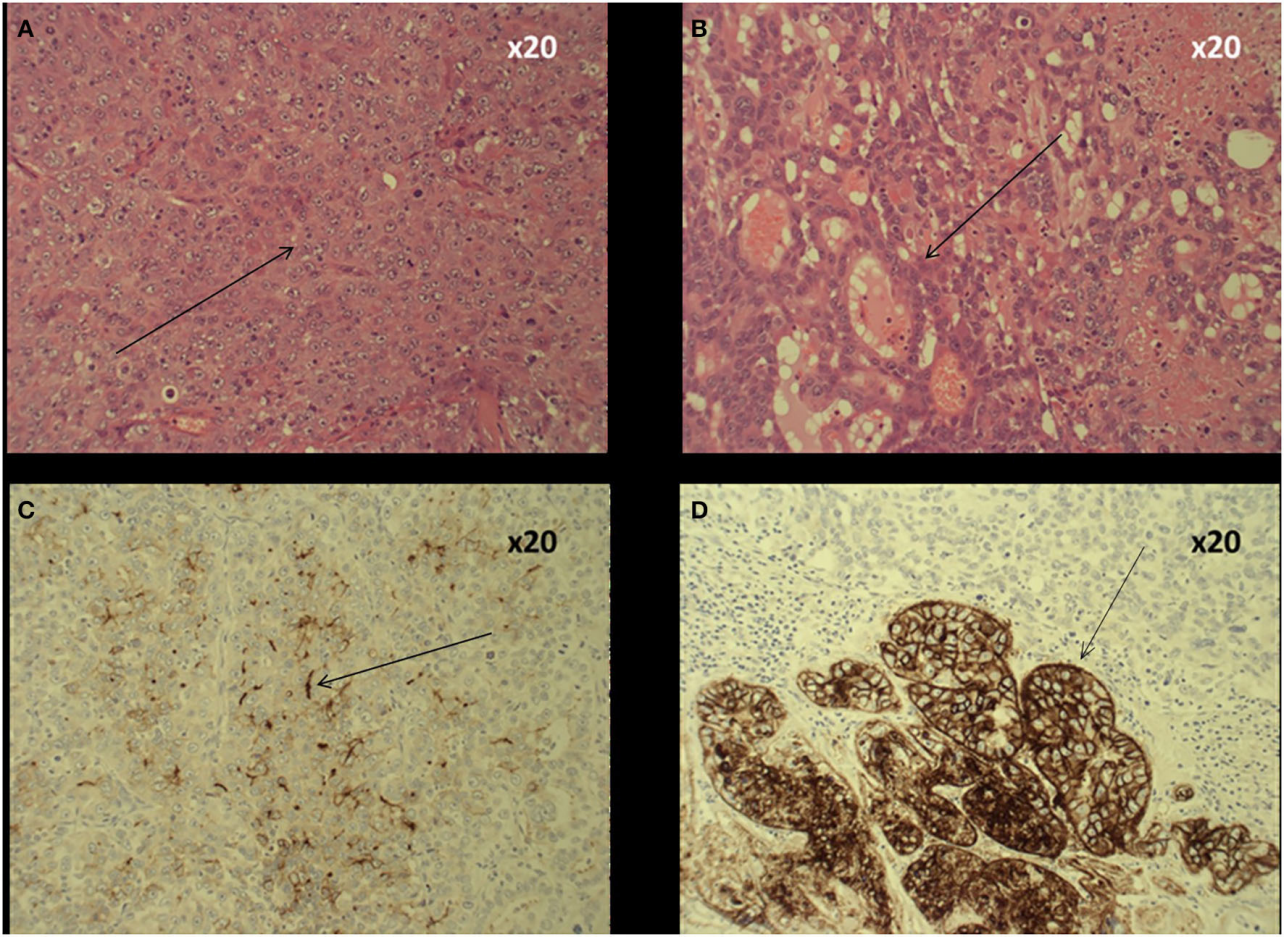
Histology of cHCC-ICC. **(A)** Haematoxylin and eosin (H&E) slide shows an area of tumor with features of poorly differentiated hepatocellular carcinoma namely nuclear pleomorphism, hyperchromasia and coarse chromatin pattern. **(B)** H&E slide showing an area within the same tumor showing more prominent glandular architecture, morphologically consistent with cholangiocarcinoma. **(C)** The area with hepatocellular morphology shows a canalicular pattern of reactivity with polyclonal CEA, supportive of hepatocellular differentiation. This area does not react with BER EP4 polyclonal antibody. **(D)** The glandular area is immunoreactive for BER EP4 supportive of glandular epithelial differentiation consistent with the cholangiocellular component.

**Figure 2:**
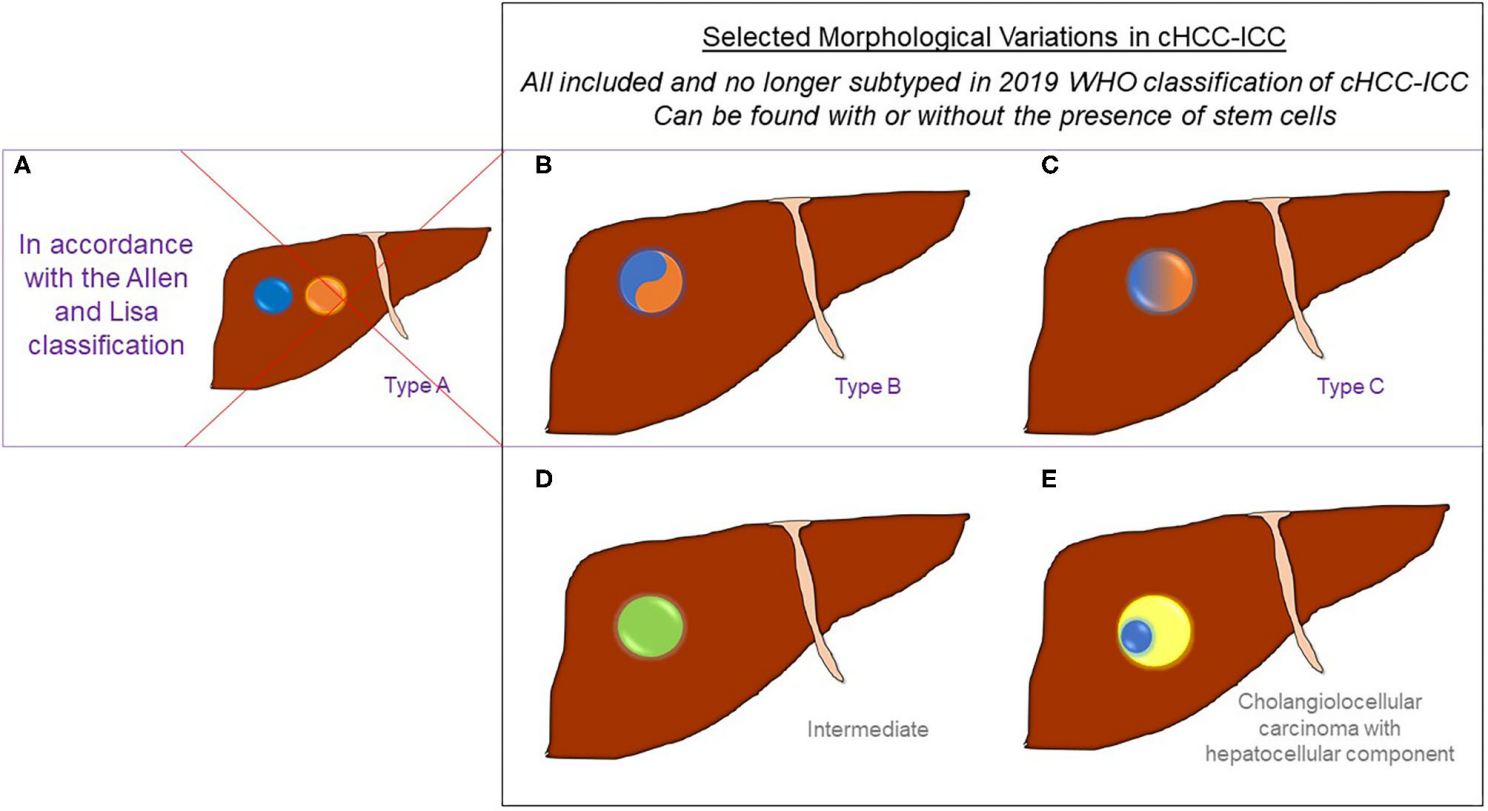
Selected Morphological Variations in cHCC-ICC. cHCC-ICC tumors contain unequivocal cells of both hepatocytic (denoted in **blue**) and cholangiocytic differentiation (denoted in **orange**) within the same tumor mass using routine hematoxylin and eosin staining. All may contain variable levels of stem cell features. Prior classification systems used to differentiate between different morphological forms of cHCC-ICC. **(A)** Represents Allen and Lisa's Type A tumors which are HCC and ICC in the same lobe of the liver but not within the same tumor mass; these are no longer diagnosed as cHCC-ICC but are considered concomitant HCC and ICC tumors (included for completeness). **(B)** Represents Allen and Lisa's Type B tumors which contain HCC and ICC histological features within the same tumor mass with a sharp transition between them. **(C)** Represents Allen and Lisa's Type C tumors show HCC and ICC histological features blending into each other within the same tumor mass. **(D)** Intermediate cell carcinoma (in **green**) **(E)** and cholangiolocellular carcinoma (in **light yellow**) but only when it also contains a hepatocellular component (in **blue)**, are also included within the definition of cHCC-ICC.

The 2019 World Health Organization (WHO) guidelines have streamlined previous histopathological classification systems (1, 3). The definition and diagnosis of cHCC-ICC now simply requires histopathological identification of unequivocal hepatocytic and cholangiocytic differentiation morphologically within the same tumor using routine hematoxylin and eosin (H&E) staining (Figure 1) (1, 3). There is no agreed proportion of each required for a diagnosis and no strict requirement to subtype the tumors (3). cHCC-ICC may or may not include cells with stem cell features, however the use of the category “cHCC-CCA with stem cell features” is no longer recommended (1, 3, 17, 18). Morphologically, the two components can be adjacent to each other or deeply intermingled, with a sharp or poorly defined transition. cHCC-ICC with a sharp, or a poorly defined transition, used to be known as type B and type C cHCC-ICC, respectively according to the 1949 Allen and Lisa classification (7). Some genomic studies still divide tumors morphologically using the Allen and Lisa classification and it is emerging that there may be significant genomic differences between them (*vide infra*) (Figure 2) (7). Rarely, cHCC-ICC may show homogenous features intermediate between hepatocytes and cholangiocytes throughout the tumor mass. This is known as “intermediate cell carcinoma of the liver” and is currently incorporated within the definition of cHCC-CCA, however there is a lack of consensus as to whether this is a distinct entity or not (1, 3, 7).

Within the cHCC-ICC tumor mass, the ICC component shows mucin-producing glandular structures within stroma, whereas HCC differentiation is characterized by Mallory-Denk bodies, bile canaliculi and a trabecular growth pattern. This can be further substantiated using a panel of immunohistochemical stains, although this is neither necessary nor sufficient for the diagnosis (Figure 1). Immunomarkers supporting cholangiocytic differentiation, include Ber EP4, MOC31, CK7, and CK19, whilst arginase-1, hep par 1 and canalicular expression of polyclonal CEA and CD10 is more supportive of hepatocellular differentiation. In the past, CK19, CD56, CD117 and nestin expression have been used to identify “stem cell” features (19). The cell of origin of at least classical cHCC-ICC could be a single form of bipotent hepatic progenitor cell capable of terminal differentiation into either hepatocytes or cholangiocytes (1, 4, 20–22).

Cholangiolocellular carcinoma (CLC) contains glandular epithelial cells consisting of thin, ductular-like structures within a dense hyalinized stroma and used to be classified as a subtype of cHCC-ICC (4, 7, 18, 23, 24). However, morphologically, this resembles ICC and CLC is now considered to be a subtype of ICC (in keeping with available genomic data), unless there is an admixed hepatocytic component (1, 3, 25).

### Imaging Characterization

**Figure 3:**
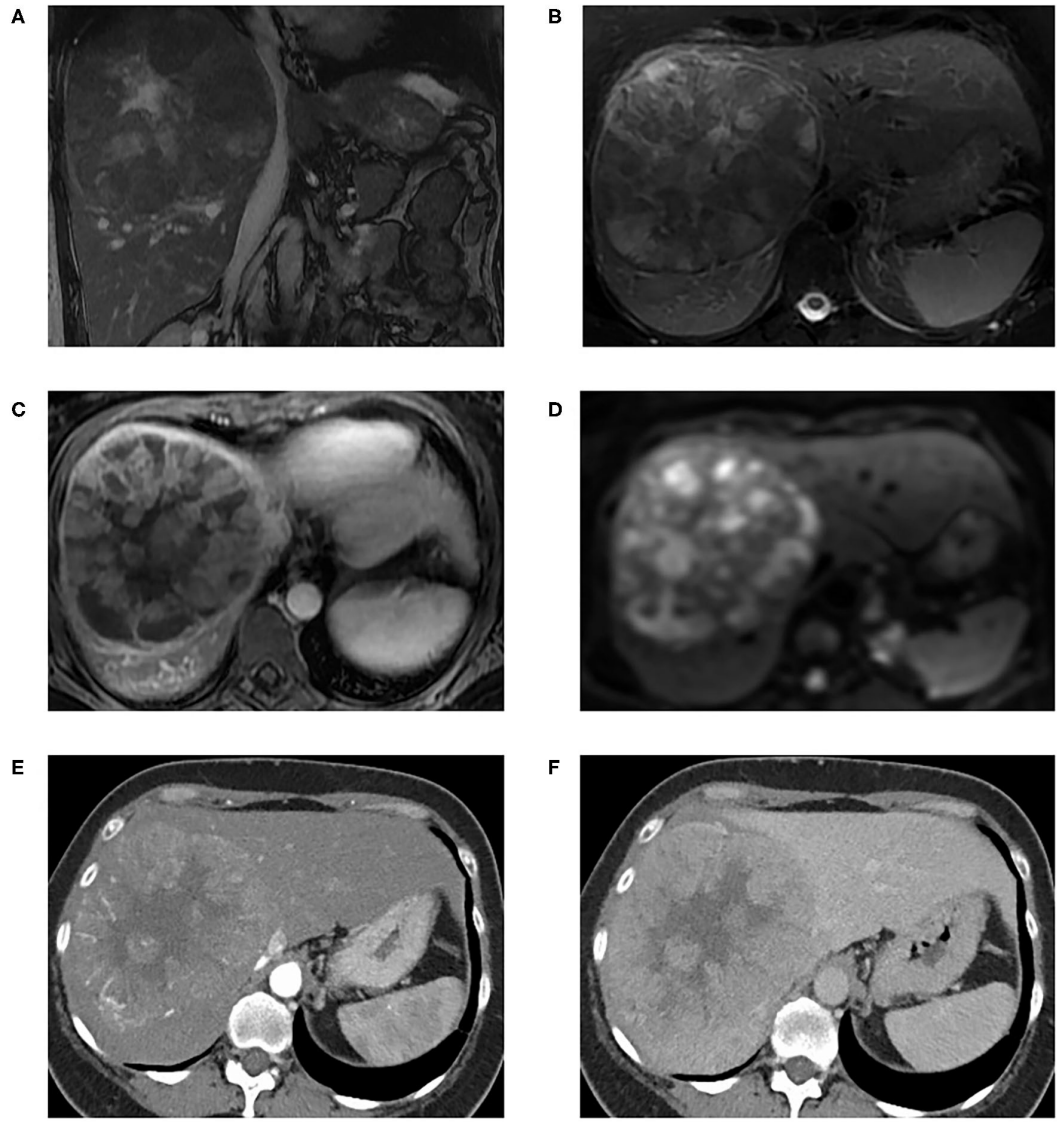
MRI liver with extracellular gadolinium contrast agent from a patient with histologically confirmed neoplastic liver lesion with variable, moderate to poor, differentiation with areas of hepatocellular differentiation and other areas of immunohistochemical evidence of cholangiocellular differentiation. Coronal FIESTA **(A)** and axial T2-weighted Fat Saturated **(B)** images show an 11cm well-defined centrally necrotic heterogeneous liver mass at the right lobe of the liver. This is effacing the IVC although there is no definite venous tumor invasion. The middle and right hepatic veins were not appreciable, presumed completely effaced; the main and branch portal veins were patent (not shown). In addition, there is associated mild intrahepatic biliary duct dilatation. T1-weighted arterial phase axial image **(C)** shows heterogeneous peripheral enhancement. Diffusion weighted imaging (*B* = 600) **(D)** shows heterogeneously restricted diffusion on correlation with Apparent Diffusion Coefficient maps. Post-intravenous contrast CT in arterial phase **(E)** and portovenous phase **(F)** for the same patient shows a large vascular mass in the central aspect of the right lobe liver with arterial hyperenhancement and portovenous wash-out, and central necrotic areas.

Cross-sectional imaging with Computed Tomography (CT) and Magnetic Resonance Imaging (MRI) are the mainstay in the characterization of liver malignancy (Figure 3) (26–31). Characteristic imaging features of HCC include arterial hyperenhancement with washout, delayed enhancing pseudocapsule, and intra-lesional fat (32–34); and those of ICC include progressive centripetal enhancement, capsular retraction, and bile duct dilatation (30). Appearances can overlap and cHCC-ICC can demonstrate features of both (30, 31, 35, 36).

The most widely adopted strategy for the diagnosis of PLC in high risk patients based on imaging alone is Liver Imaging-Reporting and Data System (LI-RADS) (34). This includes a “LR-M” category encompassing definitely or probably malignant observations which are not specifically HCC; atypical HCC, ICC and cHCC-ICC would fall into this category and a biopsy is needed (34). Features in favor of LR-M category include a targetoid mass appearance or other features such as infiltrative appearance, marked diffusion restriction, necrosis, or severe ischaemia. LI-RADS has been validated for high risk (e.g., cirrhotic) patients only and contemporary studies show the potential for misclassification of cHCC-ICC: diagnostic discordance between imaging and biopsy findings has been noted in 52% of cases of cHCC-ICC (*n* = 42) (37). Of 61 cases, 54.1% of cHCC-ICC could have been misclassified with LI-RADS using major criteria alone (35). Comparison of LI-RADS to MRI with gadolinium ethoxybenzyl diethylenetriamine (Gd-EOB) showed that ~37% of cHCC-ICC were being wrongly categorized as HCC (36). Combining imaging and biopsy (including immunophenotypical markers) can improve diagnostic performance, with a 12% increase in sensitivity reported in certain series (37).

### Circulating Tumor Markers

The diagnosis of and differentiation between HCC and ICC can be supported by circulating biomarkers (4, 38). Elevated serum Cancer Antigen 19.9 (CA19.9) is associated with ICC and elevated alpha-fetoprotein (AFP) is associated with HCC; the elevation of both or either can be seen in cHCC-ICC (4, 38). Concurrently elevated CA19.9 and AFP in a radiologically diagnosed PLC, or elevation in a biomarker discordant with the features on the imaging may indicate that the tumor is cHCC-ICC (38–40). There are several serum additional biomarkers associated with the diagnosis of HCC in particular including AFP-L3, des-γ-carboxyprothrombin (DCP), golgi protein 73 (GP73), and osteopontin (OPN), but these have not been studied robustly in appropriate series in cHCC-ICC (39, 41, 42).

## Genetic Characterization and Molecular Biology

Identification of genetic and molecular alterations in cHCC-ICC tumors may aid accurate diagnosis, define tumor etiology, support biomarker development, predict disease prognosis and guide therapy. Most studies analyze the tumor mass as a whole. However, to begin, some studies *have* looked at the distinct histological elements which resemble HCC and ICC within the cHCC-ICC tumor mass. Concordant copy number changes and shared mutations on whole exome sequencing (WES) can show that these two areas of the tumor which appear different histologically are subclones from a monoclonal origin. However, there is notable intratumour heterogeneity even in these studies, for example, there can be marked differences in the magnitude of these copy number variations and there can be key differential gene expression leading to hepatocyte-like or cholangiocyte-like differentiation, notably in *VCAN, ACVR2A*, and *FCGBP* (19, 43, 44).

Genomic studies have shown that cHCC-ICC are genetically distinct from HCC and ICC with important differences in their molecular aberrations (4, 43, 45). As initial examples, cHCC-ICC shows increased frequency of genetic alternations in RYR3 and FBN2, and increased amplifications and gains of function in MYC compared to HCC and ICC (4, 19, 46, 47). Mutations in catenin beta-1 (CTNNB1) and KRAS, commonly found in HCC and ICC respectively, have been observed at almost insignificant rates in cHCC-ICC (19). In terms of tumor suppressors, tumor protein 53 (TP53) has been consistently reported as one of the most important genes mutated in cHCC-ICC; the largest comparative genomics study to date has shown that TP53 mutations were more frequent in cHCC-ICC compared to HCC and ICC alone [49.2 vs. 31% (*p* < 0.001) and 22% (*p* < 0.0001), respectively] (19, 47).

As in HCC and ICC, non-coding alterations are common in cHCC-ICC, for example large cohorts have shown 22.9% of cases with *TERT* promoter mutations and 29.7% of cases with *NEAT1* (an intergenic non-coding RNA gene for a long non-coding RNA) alterations, but detailed comparisons to HCC and ICC and how to target these changes therapeutically are not yet clear (19, 48–50).

cHCC-ICC studies integrating both genomics and transcriptomics using RNA-seq, WES and whole genome sequencing (WGS) find similar patterns in changes of key genes and tend to find more similarities between cHCC-ICC [especially Lisa and Allen type C (poorly defined transition) cHCC-ICC] and HCC, such as in TP53 and CTNNB1, rather than ICC (even ICCs arising in cirrhotic livers). Furthermore, molecular alterations characteristically seen in ICC, such as changes in PBRM1, IDH1, IDH2, FGFR2, and BAP1 were not present across cHCC-ICC (44, 47, 51).

Transcriptomic and molecular clusters have been described in cHCC-ICC using WES techniques (44, 52). The most detailed study to date on the complex molecular profile of cHCC-ICC has been provided by an integrative genomic analysis of 133 pan-Asian cases (19). This study concluded that Allen and Lisa type B (sharply defined transition) and type C (poorly defined transition) are distinct (based on their genetic and transcriptomic data) and hence the Allen and Lisa criteria is valid on a molecular level (Figure 2) (19). The transcriptomic profile clustering in this work showed that type B cHCC-ICC was genetically more similar to ICC, with enhanced expression of biliary markers (EpCAM, KRT19, and PRDM5) and frequent KRAS and IDH1 mutations. Whereas, using similar techniques, type C cHCC-ICC was associated more closely with poorly differentiated HCC features such as increased expression of liver cell markers (APOE, GPC3 and SALL4), more frequent TP53 mutations, enrichment in immune pathways within the tumor microenvironment and raised serum AFP levels (2, 19, 53). This correlates with clinicopathological data which has shown marked similarity between type C cHCC-ICC and HCC with regards to male/female ratio, hepatitis infection, serum AFP levels and non-tumor liver histology (14, 46).

This genetic study also identified both monoclonal and multiclonal origins of the tumors irrespective of the Allen and Lisa subtype of PLC. This finding which correlates with recent studies on the trans-differentiation of hepatocytes to cholangiocytes and HCC to cHCC-ICC-like tumors, supporting the theory of plasticity of hepatobiliary cells and a critical role of the tumor microenvironment (TME) in directing the differentiation of genetically identical liver cells into different lineages (2, 19, 54–56). The dependence of tumor development on the TME is supported by the identification of associations between clinical/environmental factors and patterns of mutations in cHCC-ICC (57). To date, no data has been published for either the immune component of the TME nor tumor mutational burden in cHCC-ICC (58).

Thus, former genomic and transcriptomic studies of all cHCC-ICC subtypes disagree on the separation from HCC and/or ICC, but recent studies suggest that Type C (poorly defined transition) subtype is genetically similar to HCC, and Type B (sharply defined transition) subtype is closer to ICC (19, 59). These findings could have potential implications for therapeutic approaches e.g., type C subtype could be treated more like HCC tumors and Type B subtype could be treated like ICC. Also, the inferences from these molecular studies may have repercussions for the new simplified WHO classification which had aimed to reduce the need for morphological subtyping. The recent finding that morphological subtypes of cHCC-ICC may correlate with genomics could explain discrepancies between some studies finding genomic similarities between cHCC-ICC and HCC, and others with ICC (19).

The genomic, transcriptomic and proteomic landscape of cHCC-ICC is reliant on a small number of disparate studies with different patient cohorts internationally, which do not perfectly agree. A summary of published aberrant genomic markers (Table 1) and possible molecular drivers and targets (Table 2) therefore should be interpreted with caution. The detailed roles of oncogenic driver mutations are still poorly understood in all forms of PLC, especially cHCC-ICC. However TGF-β, Wnt, AKT, N-RAS, Notch-Hedgehog pathway activation and NF-κB pathway inactivation have all been implicated in pathogenesis, as has signaling through AXIN1, KMT2D, RB1, PTEN, FGFR, nestin, ARID1A, KEAP1, IDH1, versican, EpCAM, Erbb2, and TERT (2, 19, 47, 53, 58, 61–63). A number of these are potential drug targets being evaluated in early phase clinical trials.

**Table 1:** Genomic and molecular differences between different subtypes of primary liver cancer.

**cHCC-ICC (all forms)**		**HCC**	**ICC**
**Type B (sharply defined transition)**	**Type C (poorly defined transition)**		
Mutations in TP53 and CTNNB1 (similar to HCC) (47)		Similar mutations in TP53 and CTNNB1 (47)	
TERT promotor mutations (similar to HCC) (60)		TERT promotor mutations (60)	
Altered spectrum of target genes in the TGFβ and Wnt/CTNNB1 cell signaling pathways, and increased LEF1 and SOX9 expression tending toward biliary differentiation (similar to ICC) (52)			Altered spectrum of target genes in the TGFβ and Wnt/CTNNB1 cell signaling pathways, and increased LEF1 and SOX9 expression tending toward biliary differentiation (52)
Increased mutations of RYR3, FBN2, KNCC3, and MYC (distinct from HCC) (47)		Fewer mutations in RYR3, FBN2 and MYC (47)	
Tendency for LoH at chromosomes 3p and 14q (distinct from HCC) (46)			Tendency for LoH at chromosomes 3p and 14q (46)
Increased TP53 mutations (predominately missense) (distinct from HCC and ICC) (19)		Fewer TP53 mutations than cHCC-ICC in this study (19)	Fewer TP53 mutations (19)
Rare to have mutations in CTNNB1 (distinct from HCC and ICC) (19)		Commonly mutated CTBBN1 (19)	Commonly mutated CTBBN1 (19)
Enhanced expression of EpCAM, KRT19, and PRDM5 (19)	Increased expression of APOE, GPC3, and SALL4 (19)	Increased expression of APOE, GPC3 and SALL4 (19)	Enhanced expression of EpCAM, KRT19 and PRDM5 (19)
Frequent KRAS and IDH1 mutations (19)	More frequent TP53 mutations (19)	More frequent TP53 mutations (19)	Frequent KRAS and IDH1 mutations (19)

*Data from studies examining differences between cHCC-ICC taken as a whole tumor mass are in black, data from studies comparing Type B and C (Allen and Lisa classification) cHCC-ICC to HCC and ICC are in blue and purple, respectively (19, 45–47, 60). cHCC-ICC, combined hepatocellular-cholangiocarcinoma; HCC, hepatocellular carcinoma; ICC, intrahepatic cholangiocarcinoma; CK, Cytokeratin; CTNNB1, Catenin Beta 1; LoH, loss of heterozygosity; Rb-1, retinoblastoma (RB) Transcriptional Corepressor 1; RYR3, ryanodine receptor 3; FBN2, fibrillin 2; MYC, MYC Proto-Oncogene, BHLH Transcription Factor; EpCAM, epithelial cell adhesion molecule; KNCC3, calcium-activated potassium ion channel gene; ARID1A, AT-Rich Interaction Domain 1A; PBRM1, Polybromo 1; LEF1, lymphoid enhancer-binding factor-1; SOX9, SRY-Box Transcription Factor 9; KRT19, Keratin 19; PRDM5, PR/SET Domain 5; APOE, Apolipoprotein E; GPC3, glyican 3; SALL4, sal-like protein 4*.

**Table 2:** Summary table of potentially actionable molecular aberrations encountered in cHCC-ICC.

**Gene**	**Function**	**Frequency**	**Alteration**
Versican (VCAN)	Proteoglycan involved in cell growth, division, adhesion and migration, angiogenesis and aerobic glycolysis	21.4%	Increased frequency of mutations (usually missense) (44)
Activin A receptor type 2A (ACVR2A)	Receptor involved in cell growth and differentiation signaling	14.3%	Increased frequency of mutations (usually missense) (44)
Epithelial cell adhesion molecule (EpCAM)	Transmembrane oncogenic mediator of epithelial cell-cell adhesion, cell signaling, migration, proliferation and differentiation		Increased expression (19)
Tumor protein p53 (TP53)	Master tumor suppressor regulating cell cycle, apoptosis, senescence and DNA repair	46–57%	Higher rate of loss of function mutations (19, 44)
MYC	Oncogenic transcription factor promoting expression of factors driving cell proliferation, cell growth and cell stemness whilst inhibiting apoptosis and differentiation	73%	Higher rate of mutations and focal amplifications (19)
Telomerase reverse transcriptase (TERT)	Crucial enzymatic component of the telomerase complex that allows lengthening of DNA strand telomeres preventing apoptosis in senescent cells	19%	Higher rate of promoter mutations and focal amplifications (19)
Cyclin D1 (CCND1)	Cell cycle positive regulator with role in angiogenesis, cell migration and cell metabolism	30%	Higher rate of focal amplifications (19, 51)
Cyclin E1 (CCNE1)	Cell cycle regulator	5–24%	Higher rate of focal amplifications (19, 51)
CDK6	Cell cycle regulator	20%	Higher rate of focal amplifications (19)
Cyclin-dependent kinase N2A (CDKN2A)	Encodes for p16 and p14arf; tumor suppressor proteins that negatively regulate the cell cycle	37%	Deletions and loss of function (19)
MET	Tyrosine kinase with established oncogenic properties including activation of cancer pathways such as RAS and PI3K, cell proliferation and angiogenesis	15–24%	Higher rate of mutations and focal amplifications (19, 51)
K-RAS	GTPase protein with established oncogenic properties including activation of pathways such as MAP kinase and PI3K/mTOR pathways that promote cell growth, protein synthesis and cell division	5%	Higher rate of mutations (but lower when compared to ICC) and increased expression (19, 51)
Phosphatase and tensin homolog (PTEN)	Phosphatase acting a tumor suppressor factor via negative regulation of the Akt/PKB signaling pathway and inhibition of cell cycle and division.	10%	Higher rate of mutations (19, 51)
AT-rich interaction domain 1A (ARID1A)	Combined helicase and ATPase, part of an ATP-dependent chromatin-remodeling complex that acts as a tumor suppressor by regulating transcription of genes involved in oncogenesis	19.5%	Higher rate of mutations (19)
AT-rich interaction domain 1B (ARID1B)	Combined helicase and ATPase, part of an ATP-dependent chromatin-remodeling complex that acts as a tumor suppressor by regulating transcription of genes involved in oncogenesis	28.6%	Increased frequency of mutations (usually missense) (44)
AT-rich interaction domain 2 (ARID2)	Combined helicase and ATPase, part of an ATP-dependent chromatin-remodeling complex that acts as a tumor suppressor by regulating transcription of genes involved in oncogenesis	19.5%	Higher rate of mutations (19)
Adenomatous polyposis coli (APC)	A tumor suppressor protein regulating cell adhesion, invasion and cell proliferation by negatively regulating of beta-catenin via interaction with E-cadherin within the Wnt signaling pathway	7.2%	Increased frequency of mutations (usually missense) (44)
Retinoblastoma (RB1)	Multifunctional protein acting as a tumor suppressor by inhibiting cell cycle progression and inducing senescence thus regulating cell growth and proliferation and preventing metastasis	26%	Deletions and loss of function (19)
PTMS-AP1G1	Important component of clathrin-coated vesicles for intra-cellular transportation	11.7%	Fusion events (19)
Fibroblast growth factor receptor (FGFR)	Cell surface membrane receptor tyrosine kinase which activates secondary messanger systems key to processes such as proliferation, differentiation, cell migration, and survival	6.5%	Fusion events (19)
CTNNB1 (β-catenin 1)	Multifunctional protein involved in the regulation of gene transcription and cell-cell adhesion as part of the cadherin complex in the Wnt signaling pathway where it acts as an oncogene		Higher rate of mutations (but lower when compared to HCC) (19)
NFATC2/3	DNA-binding protein regulating cell invasiveness and migration.	7.2% (NFATC2), 28.6% (NFATC3)	Increased frequency of mutations (44)
AXIN1	Cytoplasmic protein that acts as negative regulator of the Wnt signaling pathway to induce apoptosis	25%	Deletions and loss of function
IDH1	Enzyme involved in metabolic processes that can inactivate tumor suppressor genes and activate oncogenes	21.2%	Higher rate of mutations (19)

*MYC, MYC Proto-Oncogene; BHLH, Transcription Factor; MET, MET Proto-Oncogene, Receptor Tyrosine Kinase; K-RAS, KRAS proto-oncogene, GTPase; PTMS-AP1G1, Parathymosin-AP-1 complex subunit gamma-1; CTNNB1, Catenin Beta 1; NFAT2/3, Nuclear Factor Of Activated T Cells 2/3; IDH1 gene, isocitrate dehydrogenase [NADP(+)] 1, cytosolic (2, 19, 44, 51, 53, 61)*.

### Staging and Prognosis

cHCC-ICC is staged by TNM in a clinical context (as opposed to SEER staging of epidemiological data) using the same staging algorithm as for ICC (Table 3) (64–66). It is difficult to get accurate measures of patient survival without treatment (i.e., the true prognosis) but two large epidemiological datasets from the United States provide some guidance (12, 67). Median overall survival (mOS) of patients stratified by the SEER stage for distant, regionalized, and localized cHCC-HCC was 4 months (95% CI, 3–6), 7 months (95% CI, 5–11), and 20 months (95% CI 16–28), respectively (*p* < 0.001), with the difference between regionalised and localized explained by suitability for resection (12). A similar pattern is seen using TNM staging data from the National Cancer Data Base (NCDB) where mOS was 28.6 m for patients with Stage I disease, 24.2 m for stage II, 7.5 m for stage III and 3.1 m for stage IV (67).

**Table 3:** Summary table of staging systems.

**TNM Stage**	**Tumor**	**Node**	**Metastasis**	**SEER General Staging System**
IA	T1a	N0	M0	Localized
IB	T1b	N0	M0	
II	T2	N0	M0	
				Regional
IIIA	T3	N0	M0	
IIIB	T4	N0	M0	
	Any	N1	M0	
IV	Any	Any	M1	Distant

*The tumor, node and metastasis scores of each tumor allow TNM staging. The primary tumor is classified as follows; T1 if it is a solitary tumor with no vascular invasion (T1a if ≤ 5 cm and T1b if >5 cm), T2 if it is either a solitary tumor with vascular invasion or there are multiple primary tumors (irrespective of vascular invasion), T3 if the primary tumor perforates the visceral peritoneum and, T4 if the tumor involves local extrahepatic structures by direct invasion. N1 denotes regional lymph node metastases and M1 denotes distant metastatic disease. The SEER general staging system for tumors such as cHCC-ICC is included and compared with the TNM system; some TNM stage II tumors may be classified as localized and others as regional. The majority of large epidemiological studies to date use the Surveillance, Epidemiology, and End Results (SEER) Program of the National Cancer Institute: localized cancer is limited to the anatomical site of origin without spread, regional cancer is limited to the nearby draining lymph nodes, tissues or organs by direct extension, and distant cancer has spread to distant non-continuous parts of the body (7, 10–12). SEER, Surveillance, Epidemiology, and End Results program; TNM, tumor node metastasis*.

## Treatment

In patients with localized cHCC-ICC and good performance status, surgical resection may provide the chance of long-term benefit, for example, 5 year survival rate of 30% has been reported (12, 68, 69). These tumors show locoregional spread in similar patterns to HCC (hepatic and portal venous invasion) and to ICC (lymph node dissemination). Therefore liver resection with hilar node dissection is attempted In suitable patients with satisfactory liver function, however for patients with underlying cirrhosis, resections are limited to avoid hepatic decompensation (70–72). Pre-operatively tools such as the Model for End-stage Liver Disease (MELD) score calculated from INR, bilirubin, and creatinine, can be utilized in the risk assessment to predict post-operative mortality following surgical resection (73).

The observed survival after surgery is similar to ICC, where transplant is not standard, and notably less than for HCC where transplantation may be offered (74, 75). Transplanted cHCC-ICC patients (*n* = 19) compared with transplanted HCC patients (*n* = 1147) had inferior 5-year OS rates of 48 vs. 78% (*p* = 0.01) (75). A meta-analysis of NCDB cases indicates that transplantation does not result in improved outcome when compared with resection in cHCC-ICC, making a case for careful pre-operative diagnostic assessment to minimize the risk of misdiagnosis with HCC and for the limited supply of donor livers to be more beneficially applied for conditions with better post-transplant outcomes (67, 70).

Non-surgical treatment options in patients with localized disease include ablation procedures, transarterial (chemo)embolization (TA(C)E), hepatic arterial infusional chemotherapy, radioembolization, and systemic therapy (68, 71). The data for benefit of loco-regional therapies in cHCC-ICC is limited to small retrospective studies but there are recognizable partial response rates which may allow subsequent surgical resection and potentially survival benefit (4, 68, 76, 77).

Even following treatment for localized disease it is common for the disease to recur, often with unresectable regional or distant/metastatic disease; (4, 40, 76, 78) tumor recurrence rates at 1, 3, and 5 years were 60.8, 71.8, and 80.7%, respectively in one study, and median disease-free survival of 10 months has been reported (4, 5, 69, 78). Recurrence rates seem to be non-significantly different in comparison to HCC and ICC, but mOS after recurrence tends to be worse than HCC and possibly worse than ICC (4, 5, 78).

### Systemic Treatment Options

There is no globally accepted standard first line therapy for advanced cHCC-ICC as the evidence base is limited, therefore clinicians offer first line treatments utilized for either advanced HCC or ICC to patients with Eastern Cooperative performance (ECOG) performance score (PS) 0–2. Systemic treatment planning for cHCC-ICC patients requires careful consideration of comorbid cirrhosis and compromised liver function.

Standard first line therapy for CCA is gemcitabine 1,000 mg/m^2^ and cisplatin 25 mg/m^2^ doublet chemotherapy administered on days 1 and 8 of a 21 day cycle, for patients with good ECOG PS of 0–1, based on the ABC-02 trial (79, 80). The dose of gemcitabine may be reduced to 800mg/m^2^ if there is pre-existing liver dysfunction (81–83). In terms of second-line treatments upon progression, patients may get re-treated with gemcitabine/cisplatin depending on their initial outcome, or can be referred for clinical trials. A recent trial of oxaliplatin/5-FU (mFOLFOX) plus active symptom control given to advanced biliary tract cancer (including ICC) patients after first-line gemcitabine-cisplatin doublet chemotherapy showed modest extended mOS by just under a month (5.3 vs. 6.2 months) between study arms, however differences in OS rate at 6-months (35.5 vs. 50.6%) and 12-month (11.4 vs. 25.9%) were potentially clinically meaningful (84).

For advanced HCC, cytotoxic therapies are generally not used in standard practice due to lack of efficacy and toxicity concerns, particularly in cirrhotic patients, but there is significant data to support the role of small molecule multitargeted tyrosine kinase inhibitors (TKIs) sorafenib and lenvatinib in the first-line treatment setting (85–88). More recently there has been positive data in first line treatment setting for HCC patients using immune checkpoint inhibitor (ICPI) therapies in combination with other agents such as bevacizumab, or tyrosine kinase inhibitors such as lenvatinib which has led to approval by the Food and Drug Administration (FDA) in the United States of America (89, 90). Evidence for utility of TKIs in cHCC-ICC patients is generally in the form of case-reports and single-center retrospective studies with a very weak signal of efficacy, but in the absence of international guidance and concerns about toxicity of cytotoxic chemotherapy they are commonly offered to patients (77, 91–93).

The comparative data on systemic therapy in cHCC-ICC is sparse, but tends to favor the efficacy of chemotherapy over sorafenib (77, 92, 93). In small retrospective studies (*n* = 41, 28 and 17), cytotoxic regimens seem to achieve a reasonable response rate and modest mOS benefit (77, 92, 93). In the largest of these cohorts, there were no recorded objective responses for sorafenib monotherapy (*n* = 5 evaluable), the median progression free survival (mPFS) was 4.8 m (*n* = 7) and mOS was 9.6 m (*n* = 7), whereas for gemcitabine-cisplatin doublet chemotherapy, the partial response rate was 24% (9/37 evaluable), mPFS was 8.0 m (*n* = 41), and mOS was 11.5 m (*n* = 41) (77). Another showed that both mPFS [3.0 m (95% CI, 0.0–9.1)] and mOS [10.2 m (95% CI, 3.9–16.6)] tend to be larger than observed with sorafenib [PFS 1.6 m (95% CI, 1.2–2.0), mOS 3.5 m (95% CI: 0.0–7.6)] with a statistically significantly improved hazard ratio (HR) for mOS [HR: 5.50 (95% CI, 1.17–25.84)] (92). Furthermore, on multivariate analysis, sorafenib monotherapy remained an independent poor prognostic factor for survival compared to first line gemcitabine-cisplatin chemotherapy [HR: 10.7, (95% CI, 1.4–80.7), *p* = 0.022] (92). cHCC-ICC management along the lines of ICC (chemotherapy as first line treatment) may be more effective than for HCC and should be the preferred option if safe (77, 92, 93).

Given the increasing evidence for ICPI efficacy in the management of both advanced ICC with microsatellite instability (MSI) and HCC, there is rationale to try this approach in cHCC-ICC (94, 95). A case report describing a near complete radiological response to ICPI in a cHCC-ICC patient showing no MSI but raised neoantigen burden in his tumor, has highlighted utility of this therapeutic approach in selected patients (96, 97).

### Perspectives From Pre-Clinical and Translational Studies

Improved models of cHCC-ICC may provide valuable information on neoplastic development, progression and therapeutic strategies for this rare tumor. Currently, one mouse model of cHCC-ICC has been developed (56). It was created from a mouse model of HCC by inhibiting nuclear factor kappa-B (NF-κB) signaling by deleting NF-kappa-B essential modulator (NEMO)/ nuclear factor kappa-B kinase subunit gamma (IKKγ) selectively from hepatocytes; the effect of different treatments on this model have not yet been explored (56). Patient derived organoid models of cHCC-ICC from resected combined tumors have recently been described, which demonstrate preserved histological architecture, gene expression and genomic landscape of the original tumor, permitting discrimination between different subtypes, even following long-term expansion in culture (56, 98). Drug sensitivity assays of the organoids recapitulated sensitivity to each of gemcitabine and sorafenib in one of the two cHCC-ICC models and sensitivity to sorafenib in the other (98). Sensitivity was also shown across the two models to taselisib (a beta-isoform sparing PI3K inhibitor), LGK974 (PORCN inhibitor), deltarasin (reduces KRAS activity by inhibiting KRAS-PDEδ interactions), vorinostat (HDAC inhibitor Class I, IIa, IIb, IV), SCH772984 (ERK1/2 inhibitor) (98). These models may provide a platform for drug screening and validation of “actionable” therapeutic targets in cHCC-ICC patients.

## Discussion

Given the rarity of cHCC-ICC, there are extremely limited clinical trial options available specifically for this group of patients. Genomic, pre-clinical and clinical studies underline inconsistencies between these tumors and either HCC or ICC in genotype, phenotype and treatment response, therefore it is emerging that these tumors may need to be regarded as a separate entity for optimal management. Current data supports the use of cytotoxic chemotherapy where possible for cHCC-ICC, but different histological and molecular subtypes (which is a different emphasis to the recent WHO histological guidance) could form the basis for more nuanced strategies for empirical chemotherapy, molecularly targeted treatment or immunotherapy. However, it should be noted that the current genomic, proteomic and systemic therapy evidence is underdeveloped and predominately from small, retrospective studies and more rigorous prospective data is desirable to allow more definitive conclusions. Molecular profiling and enrolment into tumor-agnostic “basket” trials selecting for molecular alterations could be helpful in the short term, to gain an understanding of how responsiveness of potentially “actionable” phenotypes may be impacted by the biology and environment of these unusual tumors. In the longer term, better pre-clinical models and international collaborations and registries with centralized pathology and radiology are highly desirable to optimize the knowledge base, and rationalize management strategies (1).

## Author Contributions

All authors made (1) substantial contributions to conception and design, acquisition of data, or analysis and interpretation of data, (2) drafting the article or revising it critically for important intellectual content, and (3) final approval of the version to be published. AA and BB: conceptualization and project administration. AA, AH, CG, AD, and BB: data curation, resources, visualization, and writing–review and editing. AA, AH, and BB: formal analysis, investigation, and validation. AA: methodology. BB: supervision. AA and AH: writing–original draft.

## Conflict of Interest

BB reported Consultancy for GenMab (paid to Institution); Advisory Boards for Roche (paid to Institution), Eisai Europe Limited (paid to Institution), research grant from Celgene Ltd (paid to Institution), Speakers Bureau for Eisai Europe Limited (paid to Institution), Travel and registration for Congress from Bayer. The remaining authors declare that the research was conducted in the absence of any commercial or financial relationships that could be construed as a potential conflict of interest.
